# *Spondias mombin* L. attenuates ventricular remodelling after myocardial infarction associated with oxidative stress and inflammatory modulation

**DOI:** 10.1111/jcmm.15419

**Published:** 2020-05-29

**Authors:** Bruna Letícia Buzati Pereira, Alexane Rodrigue, Fernanda Caroline de Oliveira Arruda, Tatiana Fernanda Bachiega, Maria Angélica Martins Lourenço, Camila Renata Correa, Paula Schmidt Azevedo, Bertha Furlan Polegato, Katashi Okoshi, Ana Angélica Henrique Fernandes, Sergio Alberto Rupp de Paiva, Leonardo Antonio Mamede Zornoff, Krista Anne Power, Marcos Ferreira Minicucci

**Affiliations:** ^1^ Internal Medicine Department Botucatu Medical School São Paulo State University (UNESP) Botucatu Brazil; ^2^ School of Nutrition Sciences Faculty of Health Sciences University of Ottawa Ottawa Canada; ^3^ Chemistry and Biochemistry Department Institute of Biosciences São Paulo State University (UNESP) Botucatu Brazil

**Keywords:** myocardial infarction, oxidative stress, *Spondias mombin*, ventricular remodelling

## Abstract

The objective of this study was to evaluate *Spondias mombin* L. (SM) pulp and its influence on cardiac remodelling after myocardial infarction (MI). Male Wistar rats were assigned to four groups: a sham group (animals underwent simulated surgery) that received standard chow (S; n = 20), an infarcted group that received standard chow (MI; n = 24), an infarcted group supplemented with 100 mg of SM/kg bodyweight/d, (MIS100; n = 23) and an infarcted group supplemented with 250 mg of SM/kg bodyweight/d (MIS250; n = 22). After 3 months of treatment, morphological, functional and biochemical analyses were performed. MI induced structural and functional changes in the left ventricle with worsening systolic and diastolic function, and SM supplementation at different doses did not influence these variables as analysed by echocardiography and an isolated heart study (*P* > .05). However, SM supplementation attenuated cardiac remodelling after MI, reducing fibrosis (*P* = .047) and hypertrophy (*P* = .006). Biomarkers of oxidative stress, inflammatory processes and energy metabolism were further investigated in the myocardial tissue. SM supplementation improved the efficiency of energy metabolism and decreased lipid hydroperoxide in the myocardium [group S (n = 8): 267.26 ± 20.7; group MI (n = 8): 330.14 ± 47.3; group MIS100 (n = 8): 313.8 ± 46.2; group MIS250: 294.3 ± 38.0 nmol/mg tissue; *P* = .032], as well as decreased the activation of the inflammatory pathway after MI. In conclusion, SM supplementation attenuated cardiac remodelling processes after MI. We also found that energy metabolism, oxidative stress and inflammation are associated with this effect. In addition, SM supplementation at the highest dose is more effective.

## 1 INTRODUCTION

Ischemic heart disease is the single most frequent cause of death worldwide.^1^, ^2^ More than 1.8 million people die each year in Europe from ischemic heart disease, mainly from myocardial infarction (MI).^1^, ^2^ MI has been defined as the focus of necrosis caused by low tissue perfusion with signs and symptoms resulting from cardiac cell death. It initiates a cascade of intracellular signalling that leads to molecular, cellular and interstitial changes that are manifested clinically as changes in size, shape and function of the heart. These complex changes characterize the cardiac remodelling process.^3^, ^4^, ^5^, ^6^ At first, cardiac remodelling is an adaptive process to maintain cardiac function. Chronically, however, with the continuity of the process, there is a progressive ventricular dysfunction and death.^3^, ^4^, ^5^, ^6^

At present, the standard treatment to prevent or at least attenuate the remodelling process after MI is reperfusion interventions and modern drug therapy.^1^, ^2^ Several drugs are associated with improved outcomes after MI, such as angiotensin‐converting enzyme inhibitors, angiotensin II receptor blockers, beta‐blockers, blockers of aldosterone, and neprilysin and angiotensin receptor inhibitors.^3^ However, because of the significant socioeconomic impact associated with the fact that long‐term mortality rates remain unacceptably high despite optimized therapy, it is relevant to identify factors that modulate the cardiac remodelling process.

As oxidative stress may play a central pathophysiological role in cardiac remodelling after MI, antioxidant supplements may be beneficial after myocardial injury.^7^ These include the supplementation of foods with antioxidant properties, such as *Spondias mombin* L. (SM). Our research group has evaluated several foods and micronutrients supplementation after an experimental model of MI. Among them, tomato, rosemary, vitamin A and zinc showed promising results.^8^, ^9^, ^10^, ^11^ In this scenario, SM could be an interesting supplement because of its unique composition, rich in antioxidant and anti‐inflammatory compounds.^7^, ^12^

SM is a natural fruit composed by a combination of vitamins, minerals, phenolic antioxidants and fibres. All parts of the plant (leaves, pulp and skin) are reported to be useful and are rich in β‐cryptoxanthin, flavonoids and phenolic compounds.^7^, ^12^ Experimental and clinical studies already showed benefits with SM supplementation or its compounds, in different contexts, such as cancer and diabetes.^7^, ^12^ In fact, Akinmoladun et al^13^ showed that supplementation with SM before cardiac injury induced by isoproterenol in rats protected against inflammation and oxidative stress. Interestingly, these effects were comparable with the effects of ramipril, an angiotensin‐converting enzyme inhibitor.^13^ Lourenço et al evaluated the influence of SM supplementation on cardiac remodelling induced by smoke exposure in rats and found that SM supplementation attenuated cardiac remodelling along with reducing oxidative stress and modulating energy metabolism.^14^ However, SM supplementation after MI was not evaluated.

Thus, the objective of this study was to evaluate SM pulp and its influence on cardiac remodelling after MI. In addition, we aimed to evaluate potential mechanisms associated with SM supplementation.

## 2 MATERIALS AND METHODS

This research protocol was approved by the Animal Ethics Committee of Botucatu Medical School (1120/2015) and was performed in accordance with the National Institute of Health's Guide for the Care and Use of Laboratory Animals. Male Wistar rats weighing 200‐250 g underwent experimental MI according to a previously described method or a simulated surgery (without coronary artery occlusion).^8^, ^9^ In brief, rats were anesthetized with ketamine (70 mg/kg) and xylazine (1 mg/kg), and after left thoracotomy, the heart was exteriorized by lateral compression of the thorax. The left atrium was retracted to facilitate ligation of the left coronary artery with wired polyvinyl (5‐0 Ethicon). The left coronary artery was ligated approximately 2 mm between the border of the left atrium and the pulmonary outflow tract. The heart was then replaced in the thorax, the lungs were inflated by positive pressure, and thoracotomy closed. After surgery, rats were housed in a temperature‐controlled room (24°C) with a 12‐hour light/12‐hour dark cycle. After 7 days, the first echocardiographic study was performed to ensure that the infarct sizes and fractional area changes (FAC) were similar between the infarcted groups before beginning treatment. After the echocardiogram, the animals were assigned to four groups: a sham group (animals underwent simulated surgery) that received standard chow (S; n = 20), an infarcted group that received standard chow (MI; n = 24), an infarcted group that received standard chow supplemented with 100 mg of SM/kg bodyweight/d (MIS100; n = 23) and an infarcted group that received standard chow supplemented with 250 mg of SM/kg bodyweight/d (MIS250; n = 22).

We selected only animals with an infarct size greater than 35% as assessed by histological analysis because we considered those with a small infarct size did not undergo cardiac remodelling.^15^ The rats were housed in individual cages in a temperature‐controlled room (24°C) with a 12‐hour light/dark cycle. Water was supplied ad libitum. Dietary intake was recorded daily. The rats were observed for 3 months, after which morphological, functional and biochemical analyses were performed.

### 2.1 Analysis of compounds and antioxidant capacity of SM pulp

The antioxidant activity was performed by the percentage of radical elimination 2,2‐ Diphenyil 1‐picryl‐hydrazyl (DPPH) in a methanol solution, as described by Brand‐Williams et al.^16^ The reduction in DPPH was followed by monitoring the decrease in its absorbance at a characteristic wavelength during the reaction.^16^

The content of the total phenolic compounds was determined using the Folin‐Ciocalteu, according Singleton et al.^17^ The sample absorbance was determined at 725 nm after 30 minutes of reaction. The calculations were performed from the standard curve and expressed in mg equivalent of gallic acid per gram of pulp.

Identification and quantification of individual polyphenolics were performed with reversed‐phase high‐performance liquid chromatography (HPLC) Ultimate 3000 BioRSDionex‐Thermo Fisher Scientific Inc, coupled to a diode array detector (DAD) and C18 column (2.0 × 50 mm, Luna (Natividade, 2013)).^18^

Total carotenoids were determined by reversed‐phase HPLC. The HPLC system was a Waters Alliance 2695 (Waters) and consisted of pump and chromatography bound to a 2996 programmable photodiode array detector, a C30 carotenoid column (3 mm, 150 × 3 × 4.6 mm; YMC) and Empower 3 chromatography data software. The HPLC system programmable photodiode array detector was set at 450 nm for carotenoids.

Carotenoids and flavonoids were quantified in one sample, in triplicate. The results were determined by averaging the peak areas on the cromatograms calibrated against known amounts of the respective standards.

Carbohydrate, protein, lipids, ashes and moisture were determined according to the American Association of Cereal Chemists methods.^19^

### 2.2 Chow preparation

The skin and pulp of SM were triturated, and the juice was maintained in a −80°C freezer. The total amount of water in the juice was determined (88.2%), and we added the equivalent of 100 mg (MIS100 group) and 250 mg (MIS250 group) of the dry extract/kg bodyweight/d to the standard chow. The doses of SM supplementations were determined according to Akinmoladun et al and adapted by our research group.^13^, ^14^ Thus, for each kilogram of standard chow, 21.5 and 54 g of the SM juice were added to the MIS100 and MIS250 groups, respectively. Using the formula described by Reagan‐Shaw et al^20^, the 100 and 250 mg of SM/kg bodyweight/d were equivalent to 329 and 610 g/d for a 60 kg human, respectively.

### 2.3 Echocardiographic analysis

After 3 months, all of the rats were weighed and evaluated by a transthoracic echocardiographic examination (General Electric Medical Systems, Vivid S6). All of the measurements were performed by the same observer blinded to the treatments and according the American Society of Echocardiography/European Association of Echocardiography.^21^

### 2.4 Isolated heart study: Langendorff preparation

After echocardiography analysis, 6‐8 animals in each group received sodium pentobarbital (50 mg/kg) and heparin (1000 UI) intraperitoneally and underwent an isolated heart study using the *Langendorff technique* following a previously described method.^11^, ^22^ Briefly, rats were subjected to sternotomy, and the aorta was dissected and cannulated. Retrograde perfusion was started with modified Krebs‐Henseleit solution. Hearts were transferred to the isolated heart study apparatus (Hugo Sachs Elektronik). A latex balloon connected to a pressure transducer was inserted in the left ventricular cavity. The volume inside the balloon was increased to change diastolic pressure from 0 to 25 mmHg. After each volume variation, we recorded diastolic and systolic left ventricular pressures, maximum left ventricular pressure decrease rate (−dp/dt max) and maximum left ventricular pressure development rate (+dp/dt max). The hearts that were used for the isolated heart study were not used for any other analysis, only for infarct size measurement, because retrograde perfusion can interfere with further biochemical analysis.

### 2.5 Morphometric analysis

The right and left ventricles (LV) (including the interventricular septum) of the remaining animals were dissected and separated. The myocyte cross‐sectional area (CSA) was determined as previously described.^10^ The lengths of the infarcted and viable muscle for both the endocardial and epicardial circumferences were determined by planimetry. Infarct size was calculated by dividing the endocardial and epicardial circumferences of the infarcted area by the total epicardial and endocardial ventricular circumferences. The measurements were performed on ventricular sections (5‐6 mm from the apex), assessed by picrosirius red staining, under the assumption that the left mid‐ventricular slice showed a close linear relationship with the sum of the area measurements from all of the heart sections.^10^

### 2.6 Lipid hydroperoxide, antioxidants and energy metabolism enzymes

Eight LV samples of each experimental group were used for measurements of the total protein and lipid hydroperoxide (LH) concentrations and enzyme activity determinations. Glutathione peroxidase (GPx, EC1.11.1.9), superoxide dismutase (SOD, EC1.15.1.1) and catalase (CAT, EC1.11.1.6) activity were assessed as previously specified.^23^, ^24^, ^25^, ^26^ Cardiac energy metabolism was assessed using β‐hydroxyacyl coenzyme‐A dehydrogenase (OHADH, EC1.1.1.35.), lactate dehydrogenase (LDH, EC1.1.1.27), citrate synthase (CS; EC4.1.3.7.), phosphofructokinase (PFK), pyruvate dehydrogenase (PD), Complex I (NADH: ubiquinone oxidoreductase), Complex II (succinate dehydrogenase) and ATP synthase (EC 3.6.3.14) activities as previously described.^23^, ^24^, ^25^, ^26^ Spectrophotometric determinations were performed with a Pharmacia Biotech spectrophotometer UV/visible Ultrospec 5000 with Swift II Application software at 560 nm. All of the reagents were purchased from Sigma).^23^, ^24^, ^25^, ^26^

### 2.7 Western blotting analysis of Nrf‐2, Keap1, SIRT1, FoxO1, ac‐FOXO, NF‐kB, phosphorylated NF‐kB (pNF‐kB), IkB‐α, IkB‐β, TNF‐α, IL‐10, IFN‐γ, and type I and III collagen

Samples of LV were homogenized in RIPA buffer and diluted in Laemmli buffer to detect SIRT1 (rabbit polyclonal IgG, sc15404; Santa Cruz Biotechnology, Inc, 1:200); Keap1 (Abcam, ab 66620, 1:2000); IL‐10 (rat monoclonal IgG, ab33471; Abcam, 1:2000); INF‐γ (mouse monoclonal IgG1, ab133566; Abcam, 1:2500); type I collagen (rabbit polyclonal IgG, sc8784R; Santa Cruz Biotechnology, Inc, 1:100); type III collagen (mouse monoclonal IgG1, ab6310; Abcam, 1:1000); TNF‐α (rabbit monoclonal IgG [rodent specific], 119485; Cell Signaling Technology, 1:200); total (mouse monoclonal IgG, sc8008, 1:200) and phosphorylated NF‐kB (rabbit monoclonal IgG, sc3302; Santa Cruz Biotechnology, Inc, 1:200); forkhead box class O (FoxO, sc11350; Santa Cruz Biotechnology, Inc, 1:100); ac‐FoxO (sc49437; Santa Cruz Biotechnology, Inc, 1:100); IkB‐α, mouse monoclonal IgG1 (sc1643; Santa Cruz Biotechnology, Inc, 1:200); and IkB‐β, mouse monoclonal IgG1 (sc74451; Santa Cruz Biotechnology, Inc, 1:100). Nuclear protein extraction from the LV was performed with a NE‐PER nuclear extraction reagent kit (Pierce Biotechnology). Nuclear extracts were used to detect Nrf‐2 (C‐20, rabbit polyclonal IgG, sc722; Santa Cruz Biotechnology, Inc, 1:400). Secondary antibodies were used according to the manufacturer's recommendations, and GAPDH (6C5), mouse monoclonal IgG1 (sc32233; Santa Cruz Biotechnology, Inc), was used for normalization.^27^, ^28^

### 2.8 Serum and cardiac values of β‐cryptoxanthin

β‐cryptoxanthin of the serum and heart was performed with HPLC. The HPLC Waters Alliance 2695 (Waters) was used and consisted of pump and chromatography bound to a 2996 programmable photodiode array detector, a C30 carotenoid column (3 mm, 150 × 3 × 4.6 mm; YMC) and Empower 3 chromatography data software. The HPLC system programmable photodiode array detector was set at 450 nm for carotenoids. The analysis of this compound was performed to confirm SM ingestion. The samples were protected from light and stored at −80°C until analysis. Regarding serum dosage, 200 μL serum aliquot was extracted with β‐cryptoxanthin using the method described by Tang et al.^29^

### 2.9 Statistical analysis

Data are expressed as the mean ± SD or as the median (lower quartile‐upper quartile). Normality was tested with the Shapiro‐Wilk test. Comparisons among the groups were performed with one‐way analysis of variance (ANOVA) complemented with the Holm‐Sidak test when the variables had normal distribution or Kruskal‐Wallis with Dunn post‐test, when the variables had non‐normal distribution. The chi‐squared test was used for mortality comparison. The Spearman trend test was used to evaluate whether the effect of SM was dose‐dependent when there was the influence of SM supplementation on the variable studied. In these analyses, the MI group was coded as 0, MIS100 as 1 and MIS250 as 2. Data analysis was performed with SigmaStat for Windows v2.03 (SPSS Inc). The significance level was 5%.

## 3 RESULTS

### 3.1 SM composition, diet and bodyweight

The SM pulp macronutrient composition is 8.72 g/100 g of carbohydrate, 0.87 g/100 g of protein, 1.51 g/100g of lipids, 0.70 g/100 g of ashes and 88.2% of moisture. The SM pulp total phenolic compounds, antioxidant activity, carotenoids and polyphenols are described in Table 1. There was no difference in the mean daily chow intake [S (n = 20): 22.1 (20.5‐23.1) g; MI (n = 24): 22.0 (19.1‐22.8) g; MIS100 (n = 23): 22.4 (20.4‐23.1) g; MIS250 (n = 22): 22.3 (19.5‐23.1) g; *P* = .847], bodyweight (*P* = .272) and mortality rate among the groups (S = 0%, MI = 12.5%, MIS100 = 17.4%, MIS250 = 18.2%; *P* = .316) throughout the study.

**TABLE 1:** Chemical composition of *Spondias mombin* L. pulp

Compounds	Value
Antioxidant activity (DPPH) (g DPPH/kg)	13.94 ± 3.0
Total phenolic compounds expressed as gallic acid (mg/100 g)	97.84 ± 20.2
Lutein (ng/100 mg)	9.77 ± 1.9
β‐cryptoxanthin (ng/100 mg)	21.02 ± 3.5
α‐carotene (ng/100 mg)	10.1 ± 1.2
β‐carotene (ng/100 mg)	13.50 ± 2.4
Gallic acid (mg/100 g)	14.9 ± 3.9
Catechin (mg/100 g)	6.0 ± 1.3
Chlorogenic acid (mg/100 g)	2.26 ± 0.8
Rutin (mg/100 g)	4.10 ± 1.8
Quercetin (mg/100 g)	3.90 ± 1.3
Luteolin (mg/100 g)	3.0 ± 0.9
3‐O‐methylquercetin (mg/100 g)	0.07 ± 0.01
Kaempferol (mg/100 g)	1.18 ± 0.9

Abbreviation: DPPH, 2,2‐ Diphenyil 1‐picryl‐hydrazyl.

### 3.2 Infarct size

**FIGURE 1:**
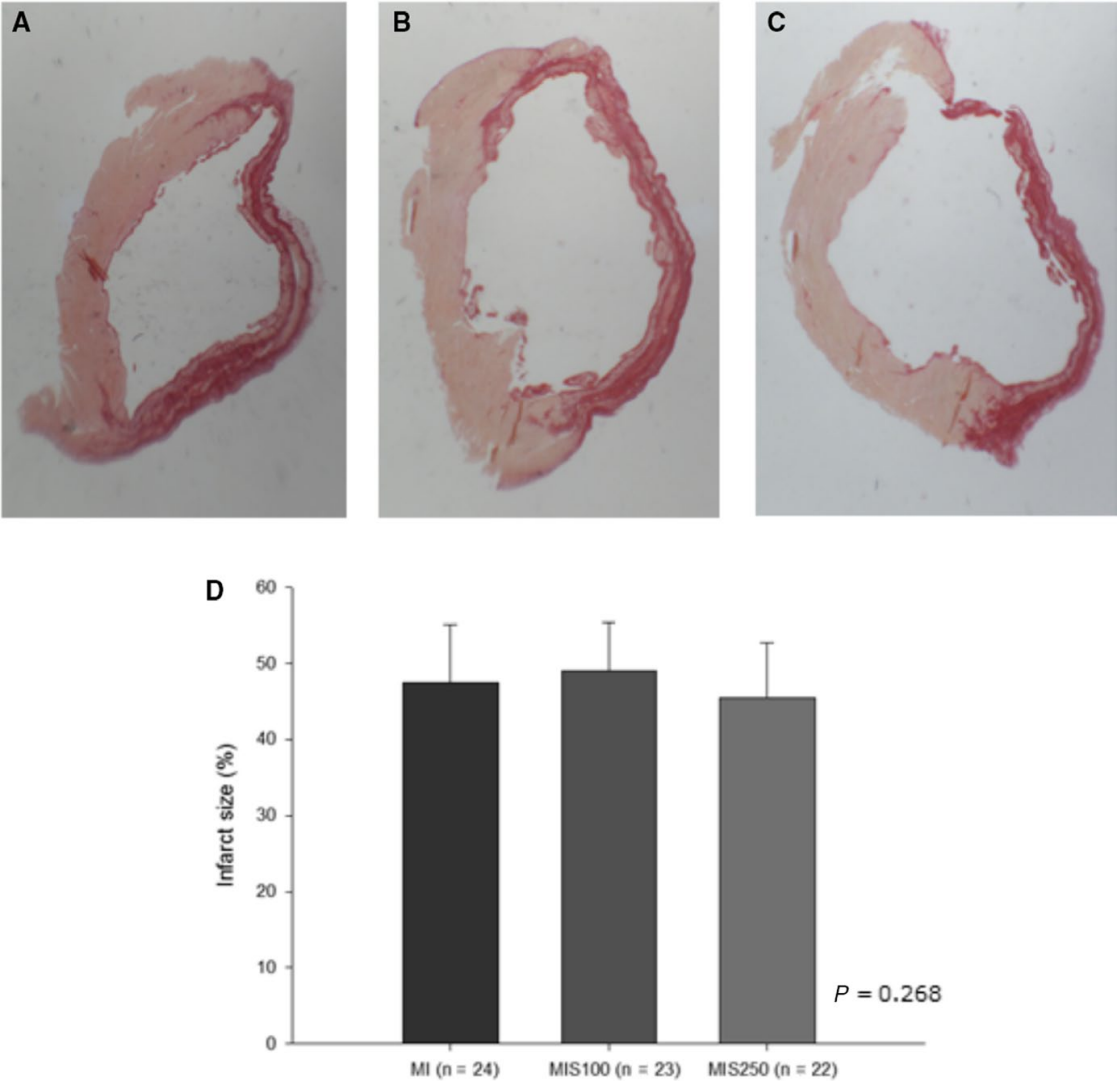
Infarct size, assessed by picrosirius red staining, 3 mo after MI. A, MI group (n = 24): 47.5 ± 1.57%; B. MIS100 (n = 23): 49.2 ± 1.32%; C. MIS250 (n = 22): 45.4 ± 1.51%; *P* = .268

The infarcted animals were allocated into three groups after the initial echocardiogram and did not present significant differences in the initial infarct size or FAC (data not shown). After euthanasia, the animals with an infarct size greater than 35% were evaluated via histology and there was no difference among the groups [MI (n = 24): 47.5 ± 1.57%; MIS100 (n = 23): 49.2 ± 1.32%; MIS250 (n = 22): 45.4 ± 1.51%; *P* = .268]. (Figure 1).

### 3.3 Bioavailablity of SM‐derived β‐cryptoxanthin

Both heart [S (n = 3): 0 (0‐0)#; MI (n = 3): 0 (0‐0)#; MIS100 (n = 5): 0.90(0.76‐0.99); MIS250 (n = 6): 1.28 (0.84‐1.93)µg/100g; *P* = .04] and serum [S (n = 3): 0 (0‐0)#; MI (n = 3): 0 (0‐0)#; MIS100 (n = 5): 0.56 (0.54‐0.69); MIS250 (n = 6): 1.31 (0.00‐0.48) µg/100g; *P* = .01] concentrations of β‐cryptoxanthin were higher in the MIS250 group compared to the sham and MI groups. The Spearman trend test showed a gradual increase in the β‐cryptoxanthin concentration, suggesting a dose‐dependent SM effect (*r* = .804; *P* = .006).

### 3.4 Morphological and functional cardiac data

The morphological and functional echocardiographic data are presented in Table 2. As expected, MI induced structural and functional changes in the LV. There was an increase in the LV systolic and diastolic areas and diameters. MI increased the AE diameter corrected by the bodyweight, LV posterior wall thickness and left ventricular mass index. Regarding the functional variables, the infarction increased IRT/RR^0.5^ and decreased FS, FAC and PWSV, indicating diastolic and systolic dysfunction. SM supplementation did not influence these variables.

**TABLE 2:** Morphological and functional data evaluated by echocardiography

Variable	S group (n = 20)	MI group (n = 22)	MIS100 group (n = 23)	MIS250 group (n = 21)	*P* value
BW (g)	437 ± 27.4	451 ± 27.7	442 ± 28.9	451 ± 27.6	.272
HR (bpm)	278 ± 41.0	280 ± 42.3	264 ± 40.6	299 ± 34.1	.064
LA/BW (mm/kg)	12.2 (11.5‐13.1)	14.9 (12.3‐18.2)^a^	14.0 (12.8‐17.2)^a^	14.0 (13.3‐16.9)^a^	<.001
LVDD/BW (mm/kg)	17.1 (16.3‐18.5)	21.9 (20.0‐24.3)^a^	21.8 (20.2‐23.9)^a^	22.2 (21.0‐23.4)^a^	<.001
LVSD/BW (mm/kg)	7.98 (7.06‐8.45)	17.6 (15.3‐18.7)^a^	16.5 (14.9‐19.6)^a^	16.7 (14.6‐19.2)^a^	<.001
PWT (mm)	1.37 (1.32‐1.42)	1.68 (1.59‐1.82)^a^	1.65 (1.51‐1.89)^a^	1.69 (1.49‐1.88)^a^	<.001
SA (cm^2^)	11.4 (9.63‐13.9)	62.9 (36.0‐73.2)^a^	59.7 (43.8‐68.4)^a^	55.7 (47.3‐73.3)^a^	<.001
DA (cm^2^)	40.9 (38.6‐43.5)	82.2 (64.7‐102)^a^	81.5 (64.5‐94.5)^a^	80.1 (66.5‐90.4)^a^	<.001
FS (%)	55.1 ± 6.73	21.7 ± 5.64^a^	22.6 ± 6.09^a^	24.0 ± 7.48^a^	<.001
PWSV (mm/s)	39.4 ± 1.18	26.0 ± 6.65^a^	26.5 ± 5.71^a^	26.2 ± 5.90^a^	<.001
Relative wall thickness	0.36 ± 0.34	0.35 ± 0.07	0.35 ± 0.05	0.34 ± 0.05	.662
LV mass index (g/kg)	1.55 (1.45‐1.77)	3.03 (2.44‐3.55)^a^	2.77 (2.41‐3.26)^a^	2.99 (2.50‐3.78)^a^	<.001
FAC	71.0 ± 7.45	35.0 ± 13.1^a^	28.9 ± 8.74^a^	28.1 ± 8.02^a^	<.001
IRT/RR^0.5^ (ms)	55.9 ± 7.24	68.7 ± 10.6^a^	65.0 ± 13.3^a^	65.1 ± 11.7^a^	.003
E/A	1.56 (1.,47‐1.80)	1.44 (1.27‐3.11)	1.49 (1.37‐2.29)	1.27 (1.18‐6.06)	.904
EDT (ms)	49.0 (41.0‐52.0)	45.0 (34.5‐49.0)	44.0 (39.3‐56.3)	37.0 (37.0‐47.3)	.114

Note

One‐way ANOVA/Holm‐Sidak; Kruskal‐Wallis/Dunn.

Data are expressed as the mean ± SD or as the median (lower quartile‐upper quartile).

Abbreviations: BW, bodyweight; DA, diastolic area; E/A, peak velocity of early ventricular filling/peak velocity of transmitral flow during atrial contraction; EDT, E wave deceleration time; FAC, fractional area change; FS, endocardial fractional shortening; HR, heart rate; IRT/RR^0.5^, isovolumetric relaxation time adjusted by heart rate; LA, left atrium; LVDD, left ventricular end diastolic diameter; LVSD, LV end systolic diameter; PWSV, posterior wall shortening velocity; PWT, LV posterior wall thickness; SA, systolic area.

^a^ *P* < .05 vs sham.

The isolated heart analysis data are presented in Table 3. MI significantly reduced the developed pressure and both the maximum rate of ventricular pressure rose and decreased (+dp/dt max and −dp/dt max), suggesting worsening of both systolic and diastolic function. However, SM supplementation did not alter these functional variables.

**TABLE 3:** Isolated heart data

Variable	S group (n = 6)	MI group (n = 7)	MIS100 group (n = 6)	MIS250 group (n = 8)	*P* value
+dp/dt max (mmHg/s)	2625 (262‐107)	1517 (647‐244)^a^	1541 (579‐236)^a^	1296 (647‐228)^a^	.002
−dp/dt max (mmHg/s)	1812 (259‐105)	1089 (412‐156)^a^	1104 (436‐178)^a^	937 (467‐165)^a^	.004
DP (mmHg)	105.8 ± 20.2	63.6 ± 20.5^a^	68.3 ± 21.5^a^	52.2 ± 24.4^a^	.001

Note

One‐way ANOVA/Holm‐Sidak; Kruskal‐Wallis/Dunn.

Data are expressed as the mean ± SD or as the median (lower quartile–upper quartile).

Abbreviations: +dp/dt max, maximum rate of ventricular pressure rise; DP, developed pressure; −dp/dt max, decreased maximum rate of ventricular pressure rise.

^a^ *P* < .05 vs sham.

**FIGURE 2:**
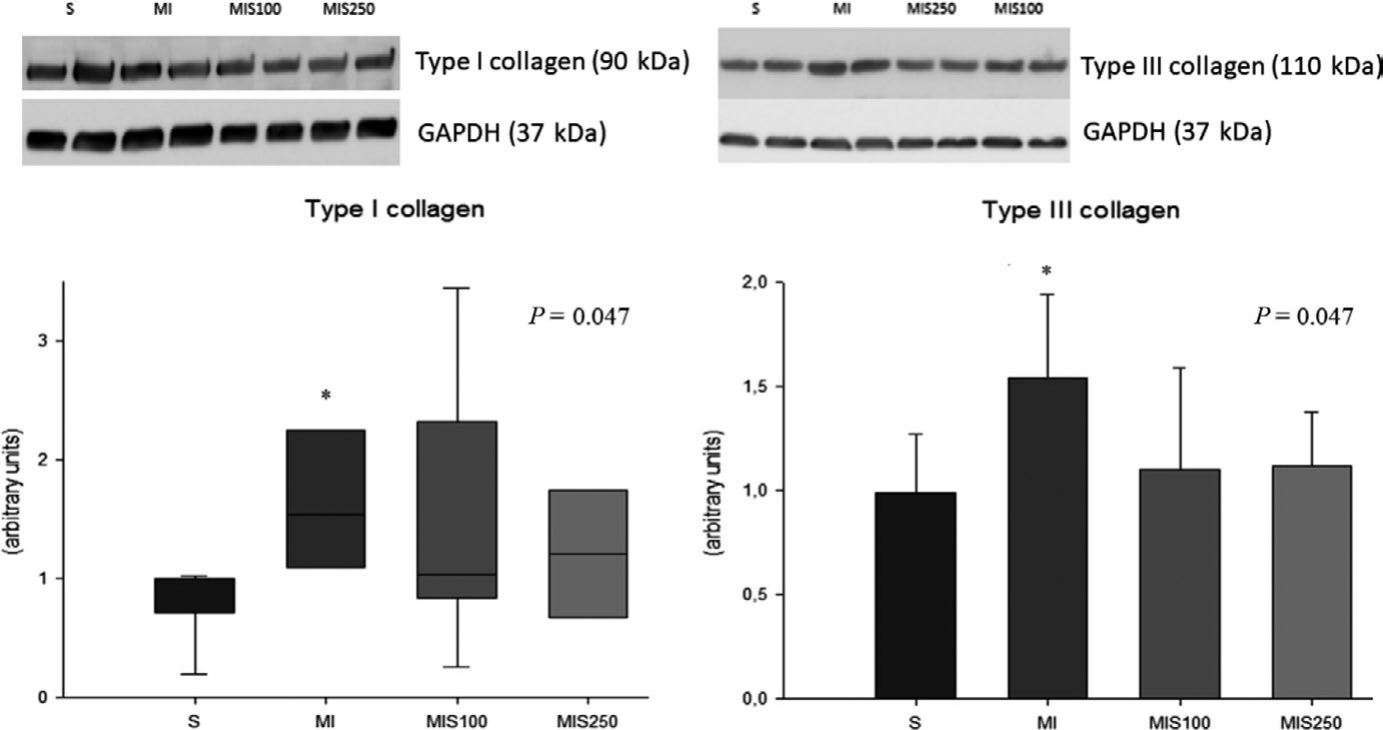
Left ventricle type I and III collagen expression (representative blots; eight animals per group)

SM supplementation attenuated hypertrophy and fibrosis induced by MI. The CSA in the MIS250 group was lower than in the MI group, and the MI group had higher values than the S group [S (n = 6): 190.5 ± 11.2; MI (n = 6): 201.9 ± 5.24; MIS100 (n = 6): 197.8 ± 6.09; MIS250 (n = 6): 190.4 ± 5.06 µm^2^ (*P* = .006)]. In addition, the MI group had higher expression of type I and III collagen compared to the sham and supplemented groups (Figure 2).

Thus, the morphological variables suggested that SM attenuated the ventricular remodelling process induced by MI. To investigate the mechanisms associated with SM supplementation, we investigated energy metabolism, oxidative stress and inflammatory pathways in myocardial tissue.

### 3.5 Cardiac inflammatory process

**FIGURE 3:**
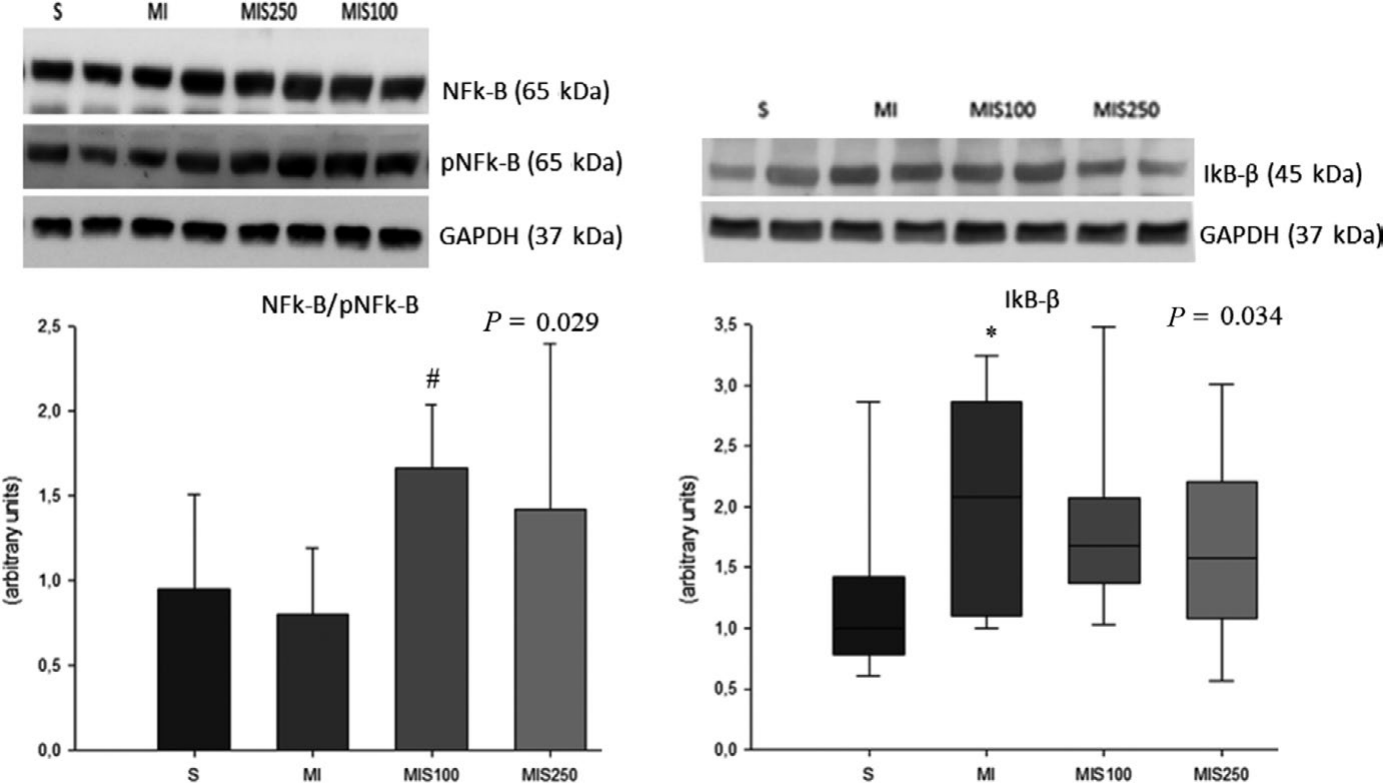
Left ventricle expression of NF‐kB/pNF‐kB and IκB (representative blots; eight animals per group)

**FIGURE 4:**
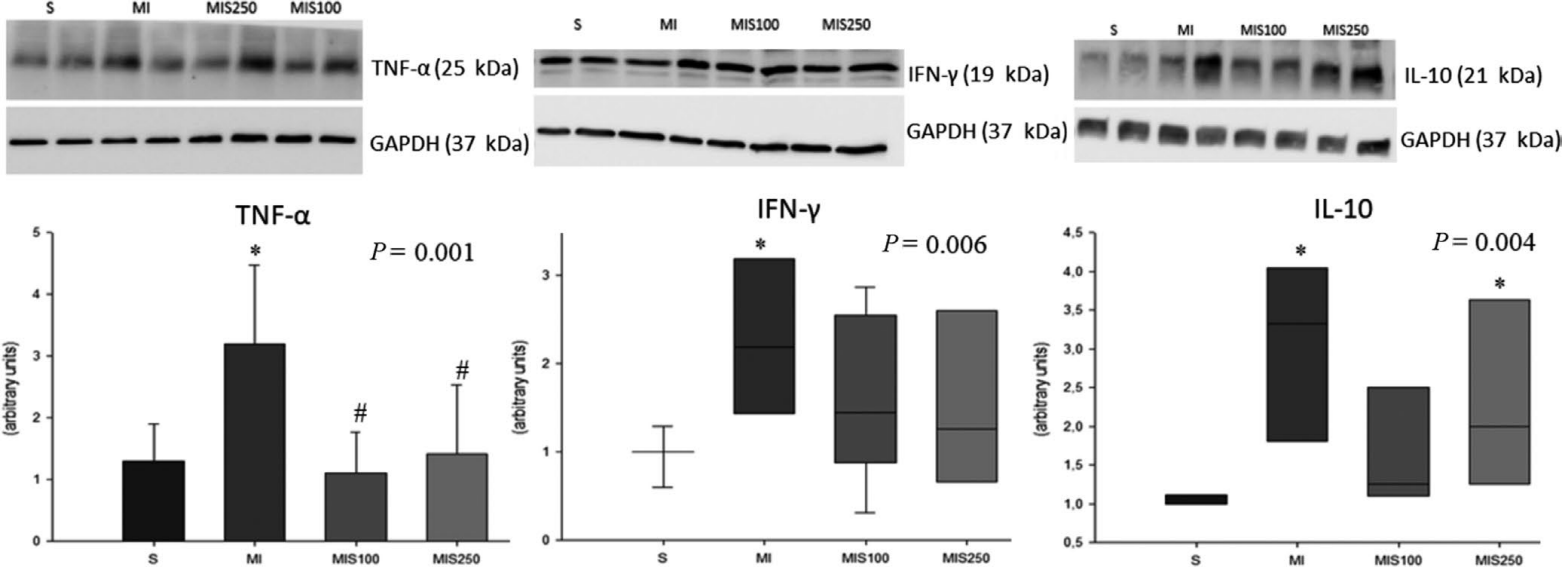
Left ventricle expression of TNF‐α, IFN‐γ, and IL‐10 (representative blots; eight animals per group)

Regarding the inflammatory process, the infarcted group presented higher IκB‐β expression than the sham group, and the MIS100 group showed higher expression of the total NF‐kB/phosphorylated ratio than the MI group, suggesting that supplementation attenuated the activation of the inflammatory pathway (Figure 3). Likewise, SM supplementation reduced inflammation caused by MI assessed by the expression of inflammatory cytokines. In fact, the MI group presented higher expression of TNF‐α than both the sham group and the infarcted groups supplemented with SM. It is also interesting that the Spearman trend test showed a gradual decrease in TNF‐α expression, suggesting a dose‐dependent SM effect (*r* = −0.494; *P* = .023). MI also presented higher interferon gamma expression than the sham group. In relation to interleukin 10, the MI and MIS250 groups had higher cytokine expression levels than the sham group (Figure 4).

### 3.6 Cardiac oxidative stress

Myocardial infarction increased oxidative stress as observed by increased LH concentration and reduced activity of the antioxidant enzymes. SM supplementation was effective in improving oxidative stress by attenuating the formation of LH. SM supplementation also attenuated the reduction in CAT and GPx. The higher dose of supplementation was more effective. SM supplementation at the highest dose attenuated the reduction in SOD (Table 4). However, no significant differences in protein expression were found via Western blotting for SIRT1, Keap1 and Nrf‐2 in the ratio between total and acetylated FoxO1 and IkB‐α normalized by GAPDH (Figures S1 and S2).

**TABLE 4:** Oxidative stress and energy metabolism in heart tissue

Variable	S group (n = 8)	MI group (n = 8)	MIS100 group (n = 8)	MIS250 group (n = 8)	*P* value
Superoxide dismutase (nmol/mg tissue)	14.1 ± 1.70	11.28 ± 0.94^a^	11.2 ± 1.58^a^	12.9 ± 1.88	.003
Catalase (nmol/mg tissue)	57.6 (45.3‐65.8)	36.6 (33.1‐42.7)^a^	36.6 (33.1‐43.6)	39.2 (33.1‐40.9)	.017
Glutathione peroxidase (nmol/mg tissue)	41.3 ± 5.63	24.6 ± 3.62^a^	32.7 ± 6.55	39.4 ± 9.71^b^	<.001
Lipid hydroperoxide (nmol/mg tissue)	267.26 ± 20.7	330.14 ± 47.3^a^	313.8 ± 46.2	294.3 ± 38.0	.032
LDH activity (nmol/mg protein)	73.2 ± 15.9	90.2 ± 16.9	83.4 ± 13.4	84.1 ± 10.1	.169
PFK (nmol/g tissue)	256.3 ± 34.0	327.0 ± 69.6	216.6 ± 65.0^b^	266.9 ± 82.7	.022
PDH (nmol/g tissue)	275.8 ± 31.2	204.0 ± 58.2^a^	248.6 ± 43.2	207.5 ± 32.7^a^	.008
CS activity (nmol/ mg protein)	26.7 ± 3.19	17.5 ± 5.64^a^	13.0 ± 3.09^a^	17.8 ± 3.63^a^	<.001
OHADH activity (nmol/mg protein)	23.6 (20.3‐25.0)	8.90 (8.15‐10.4)^a^	8.96 (8.67‐9.21)^a^	16.7 (15.3‐19.2)	<.001
ATP synthase activity (nmol/mg tissue)	30.0 (24.0‐32.1)	17.5 (15.0‐20.4)^a^	17.8 (16.7‐24.3)^a^	20.6 (19.8‐24.7)	.001
Complex I activity (nmol/mg tissue)	7.45 ± 1.40	5.37 ± 1.45^a^	4.63 ± 1.04^a^	5.18 ± 0.90^a^	<.001
Complex II activity (nmol/mg tissue)	4.34 ± 0.90	2.56 ± 0.60^a^	2.23 ± 0.56^a^	2.30 ± 0.37^a^	<.001

Abbreviations: ATP synthase activity; CS activity, citrate synthase activity; LDH activity, lactate dehydrogenase activity; OHADH activity, 3‐hydroxyacyl coenzyme‐A dehydrogenase activity; PDH, pyruvate dehydrogenase; PFK, phosphofructokinase.

^a^ *P* < .05 vs sham.

^b^ *P* < .05 vs MI.

### 3.7 Cardiac energy metabolism

The data on the enzymes of cardiac energetic metabolism are presented in Table 4. Infarction increased the activity of PFK. In this scenario, the group supplemented with the lowest dose of SM was different from the MI group. Infarction decreased the activity of citrate synthase and reduced the activity of complexes I and II, and the supplementation did not interfere in this variable. Infarction decreased the activity of the PD complex, and the lowest dose of SM supplementation attenuated this reduction. Infarction decreased the activity of ATP synthase and β‐hydroxyacyl coenzyme A dehydrogenase and the highest dose of SM supplementation attenuated this reduction. The Spearman trend test showed a gradual increase in ATP synthase activity, suggesting a dose‐dependent SM effect (*r* = .468; *P* = .021).

## 4 DISCUSSION

The objective of this study was to evaluate the influence of SM supplementation on cardiac remodelling after MI. Our data showed that different doses of SM supplementation attenuated the cardiac remodelling process after MI through reduction in fibrosis, hypertrophy, improvement of oxidative stress, energy metabolism and inflammatory activity.

We must highlight that the infarct size was similar between the groups. Thus, this variable associated with worse outcomes after MI did not influence our results. It is also important to mention that SM supplementation was effective at increasing serum and cardiac levels of β‐cryptoxanthin, the major carotenoid present in SM pulp.^7^ In addition, the analysis of SM pulp showed high levels of phenolic acids and flavonoids compounds, characterizing this fruit as having great anti‐inflammatory and antioxidant properties.

It is important to note that MI induced, as expected, LV morphological and functional changes. Although SM supplementation did not influence echocardiographic and isolated heart variables, it reduced the CSA and types I and III collagen following MI. LV chamber enlargement, cardiac fibrosis and myocyte hypertrophy are the most important morphological changes in this experimental model of MI.^3^, ^4^, ^5^, ^6^ Therefore, we conclude that SM supplementation attenuated cardiac remodelling after MI. We also believe that supplementation of higher doses of SM or during a longer period of time after MI could improve diastolic or systolic dysfunction.

The regulation of cardiac metabolism is complex, and lipids are involved in this process. In the heart, fatty acids are the main energy substrate used by the mitochondria to deliver energy to the myocytes.^30^, ^31^ β‐hydroxyacyl coenzyme A dehydrogenase is one of the enzymes responsible for the oxidation of fatty acids in acetyl‐CoA, which in the citrate cycle will form citric acid by citrate synthase and generates NADH and FADH_2_ for the electron transport chain. Under aggression conditions, the main energy substrate is replaced by glucose, which needs less oxygen to generate energy. However, it generates less ATP than fatty acid oxidation.^30^, ^31^ In our study, the infarcted animals presented lower activity of the enzymes PDH, β‐hydroxyacyl coenzyme A dehydrogenase, citrate synthase, complex I, complex II and ATP synthase, suggesting that there was a reduction in energy production after infarction from both energy sources.

However, 100 mg of SM supplementation increased PDH and the decreased PFK activity, and the higher dose of SM increased the activity of β‐hydroxyacyl coenzyme A dehydrogenase and ATP synthase. Thus, enhancement of energy metabolism, which may improve energetic efficiency and restore the generation of energy, is a potential mechanism that has been modulated by SM supplementation.

Importantly, other mechanisms associated with cardiac remodelling, beyond energetic metabolism, were also investigated, such as the inflammatory process and oxidative stress. Pro‐inflammatory cytokines such as TNF‐α, and IFN‐γ promote structural and functional changes in the heart with consequent worsening of heart failure.^32^, ^33^ It is interesting to note that they can also stimulate reactive oxygen species (ROS) synthesis. Likewise, NF‐kB is the main transcriptional factor that stimulates the synthesis of inflammatory cytokines. In unstimulated cells, the NF‐kB proteins are located in the cell cytoplasm, associated with a family of inhibitor proteins known as IκB (eg IκB‐α and IκB‐β). When this pathway is stimulated, IκB is degraded and NF‐kB translocates to the nucleus where it binds to DNA and stimulates the transcription of proteins such as cytokynes.^32^, ^33^, ^34^ In addition, NF‐kB can be extensively modified through post‐translational modifications that also influence its activity. In our study, SM supplementation decreased the phosphorylation of NF‐κB compared to the infarcted group. In addition, the infarcted group increased the expression of IκB‐β expression compared to sham group, and groups supplemented with SM had similar values compared to the sham group. Therefore, our results suggest that supplementation of SM in different doses reduced the inflammatory process after MI.

Despite these results, the expression of SIRT1, a protein whose activation may participate in cardioprotection regulating processes of inflammation and oxidative stress, showed no difference between the groups.^35^ SIRT1 controls the acetylation of proteins such as the FoxO and NF‐kB, inhibiting their transactivation in response to oxidative stress.^34^ We also did not find differences between the groups in the expression of total and acetylated FoxO1; perhaps another inflammatory pathway, not analysed in the present work, could be activated.

The cellular oxidative state is determined by the amount of ROS produced endogenously by the cell and by the effectiveness of the antioxidant systems. Several cellular systems produce ROS during normal cell metabolism.^36^, ^37^ To maintain normal levels of these species, cells have antioxidant mechanisms. The first line of defence is composed of the enzymes SOD, GPx and CAT, which reduce ROS until the formation of water. When there is an imbalance between the ROS production and the activity of the antioxidant systems, which favours the increase of ROS, the cell is under oxidative stress.^36^, ^37^ ROS are important mediators of physiological functions, but in higher concentrations, they can modify the cardiac metabolism, triggering systolic and diastolic function impairment.^36^, ^37^ In our study, infarction increased oxidative stress by increasing the cardiac concentrations of LH and reducing the activity of GPx and SOD, and SM supplementation was effective at improving oxidative stress by attenuating the formation of LH and attenuating the reduction in antioxidant enzymes. However, there was no influence in the Nrf2/Keap1 pathway, which can be activated in response to oxidative stress. On the other hand, studies have shown that different antioxidant signalling pathways are activated depending on the agent inducing oxidative stress, intensity and duration, and cell type and their physiological state.^38^

Few studies have evaluated the effects of SM on the cardiac remodelling process. SM supplementation before cardiac injury induced by isoproterenol showed that SM supplementation modulates oxidative stress and inflammation.^13^ Likewise, in the model of cardiac remodelling induced by exposure to tobacco smoke (ETS) in rats, SM supplementation attenuated the higher values of left cardiac chamber diameters, left ventricular mass index and the myocyte CSA. In addition, SM supplementation decreased the higher cardiac levels of LH induced by ETS, but had no effects in the inflammatory process.^14^ Our results after MI reinforce the beneficial effects of SM supplementation on the cardiac remodelling process and highlight that foods with antioxidant and anti‐inflammatory profiles are important areas of research. In addition, the higher dose of supplementation showed better results after MI. To the best of our knowledge, this is the first study to investigate the effects of SM supplementation after MI.

Finally, regarding our results, we must understand some apparently discrepant results. In this sense, the temporal evolution of the remodelling process is well established. Thus, remodelling begins with an injury to the heart. In response to this aggression, myocardial genetic, molecular and cellular changes can occur. This phenomenon, in turn, results in deleterious cardiac responses in different systems, including alterations in immune and inflammatory systems, cell death, collagen accumulation, energy metabolism deficit and oxidative stress. As the process evolves, all of these alterations will induce changes on cardiac morphological and functional variables. Therefore, with a longer follow‐up time, we believe that the beneficial biochemical and morphological changes induced by SM supplementation in this study would result in cardiac functional improvement.^3^, ^4^, ^5^, ^6^

SM supplementation attenuated the cardiac remodelling process after MI through the reduction in fibrosis, hypertrophy, and improvement of oxidative stress, energy metabolism and inflammatory activity. In addition, SM supplementation at the highest dose is more effective.

## CONFLICT OF INTEREST

The authors confirm that there is no conflict of interest.

## AUTHOR CONTRIBUTIONS

**Bruna Letícia Buzati Pereira** involved in conceptualization, formal analysis; investigation and writing of the original draft. **Alexane Rodrigue**, **Fernanda Caroline de Oliveira Arruda**, **Tatiana Fernanda Bachiega**, **Maria Angélica Martins Lourenço** and **Paula Schmidt Azevedo** involved in investigation and writing of the original draft. **Camila Renata Correa** involved in investigation, methodology and writing of the original draft. **Bertha Furlan Polegato**, **Katashi Okoshi** and **Ana Angélica Henrique Fernandes** involved in investigation, methodology and writing of the original draft. **Sergio Alberto Rupp de Paiva** and **Leonardo Antonio Mamede Zornoff** involved in conceptualization, methodology and writing, reviewing and editing of the manuscript. **Krista Anne Power** involved in investigation, methodology and writing, reviewing and editing of the manuscript. **Marcos Ferreira Minicucci** involved in conceptualization, methodology, formal analysis, funding acquisition and writing, reviewing and editing of the manuscript.

## Author Contribution

**Bruna LB Pereira:** Conceptualization (equal); Formal analysis (equal); Investigation (lead); Writing‐original draft (lead). **Alexane Rodrigue:** Investigation (equal); Writing‐original draft (equal). **Fernanda Arruda:** Investigation (equal); Writing‐original draft (equal). **Tatiana F Bachiega:** Investigation (equal); Writing‐original draft (equal). **Maria AM Lourenço:** Investigation (equal); Writing‐original draft (equal). **Camila Correa:** Investigation (equal); Methodology (equal); Writing‐original draft (equal). **Paula S Azevedo:** Investigation (equal); Writing‐original draft (equal). **Bertha F Polegato:** Investigation (equal); Methodology (equal); Writing‐original draft (equal). **Katashi Okoshi:** Investigation (equal); Methodology (equal); Writing‐original draft (equal). **Ana AH Fernandes:** Investigation (equal); Methodology (equal); Writing‐original draft (equal). **Sergio AR Paiva:** Conceptualization (equal); Methodology (equal); Writing‐review & editing (equal). **Leonardo AM Zornoff:** Conceptualization (equal); Methodology (equal); Writing‐review & editing (equal). **Krista Power:** Investigation (equal); Methodology (equal); Writing‐review & editing (equal). **Marcos F Minicucci:** Conceptualization (lead); Formal analysis (equal); Funding acquisition (lead); Methodology (equal); Writing‐review & editing (equal). 

## Supporting information

Fig S1

Fig S2

## Data Availability

The data that support the findings of this study are available from the corresponding author upon reasonable request.
