# Supplementary figures and images for: Spondias mombin L. attenuates ventricular remodelling after myocardial infarction associated with oxidative stress and inflammatory modulation
Source: J Cell Mol Med. 2020 May 29;24(14):7862–72. doi: 10.1111/jcmm.15419 (PMC7348186; doi:10.1111/jcmm.15419)

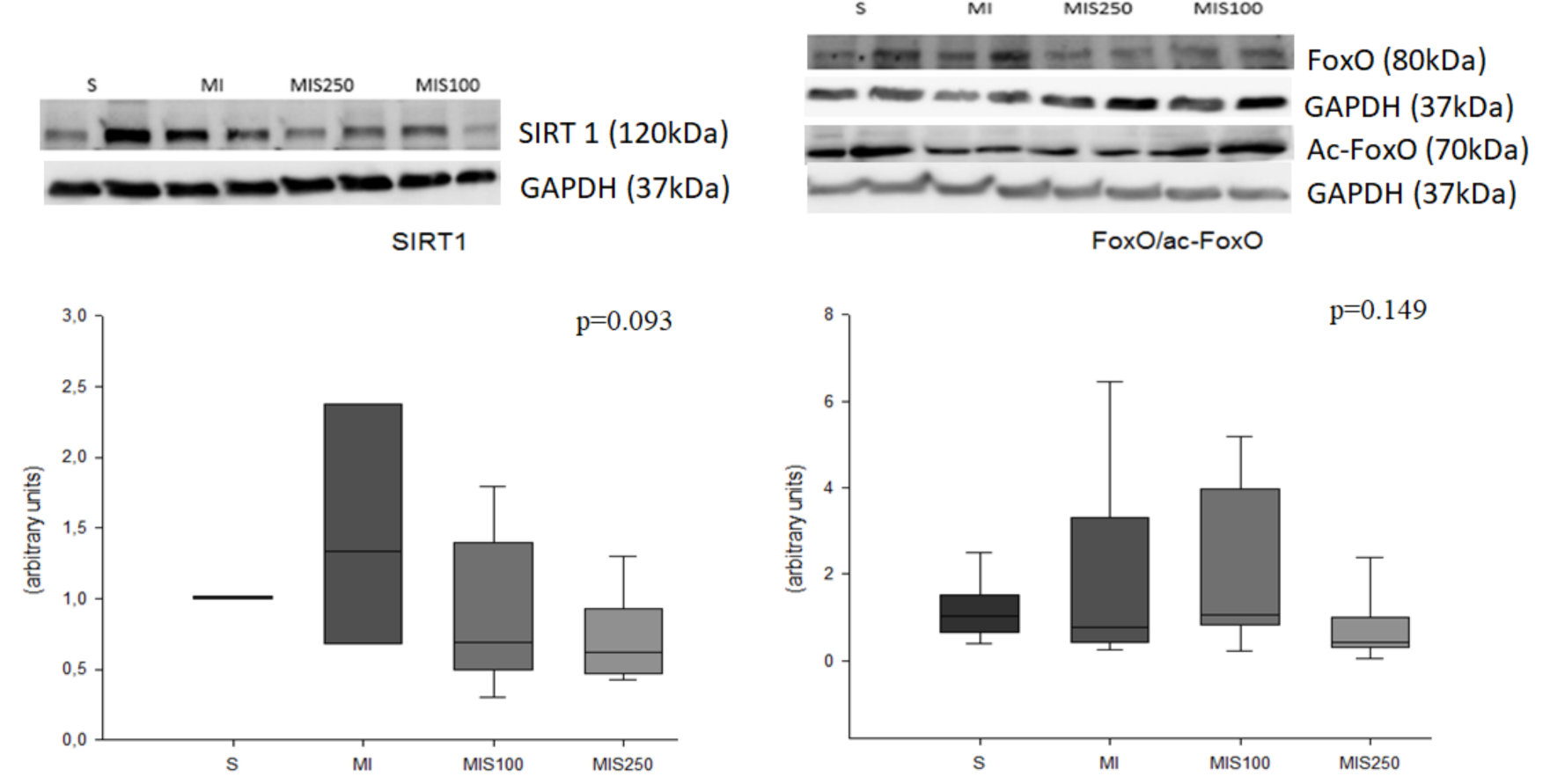

Supplement: Supplementary file 1 — Fig S1 [file JCMM-24-7862-s001.tif]

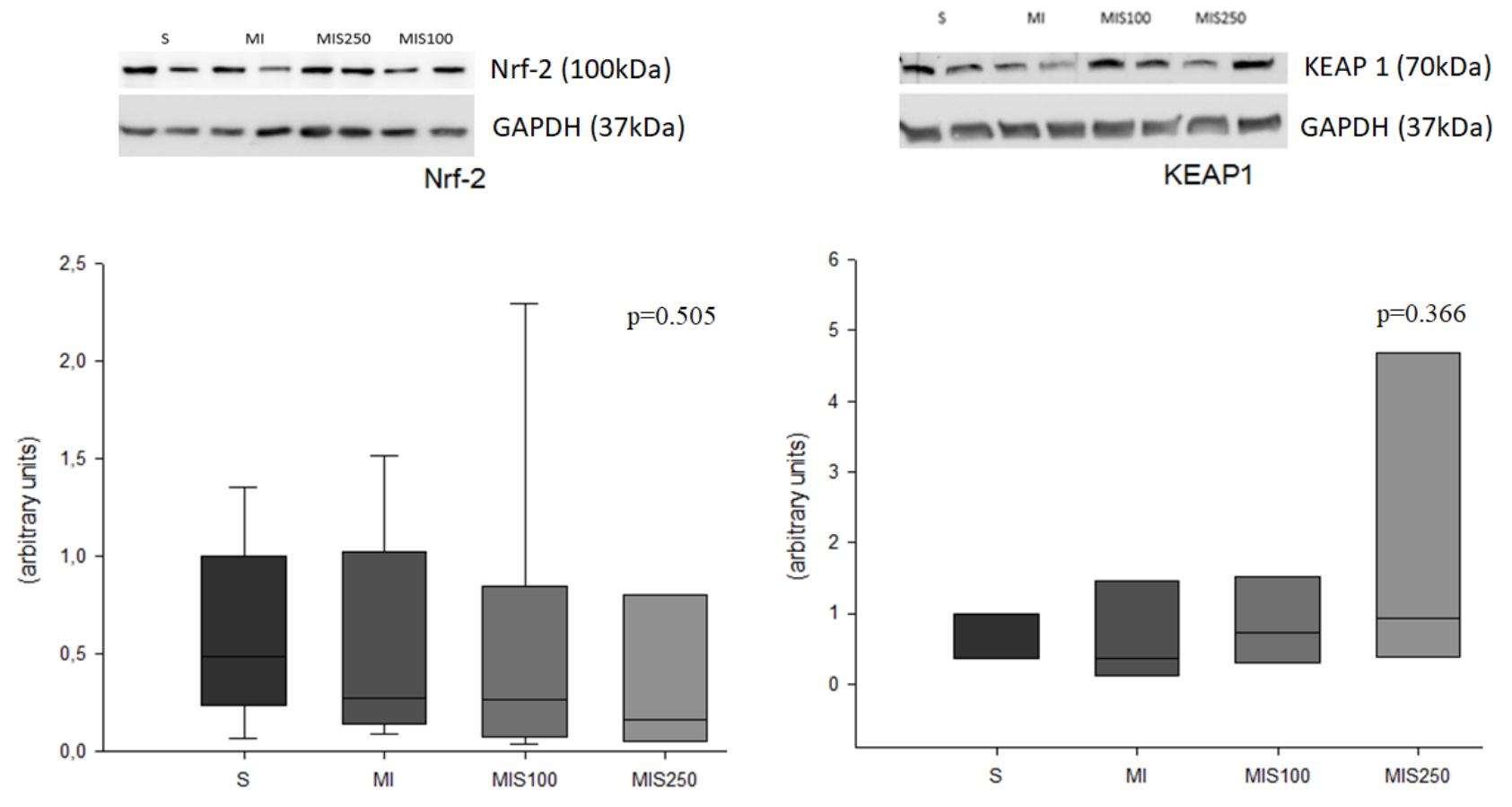

Supplement: Supplementary file 2 — Fig S2 [file JCMM-24-7862-s002.tif]
